# Supplementary material for: State-Level Health Disparity Is Associated with Sarcoidosis Mortality
Source: J Clin Med. 2021 May 27;10(11):2366. doi: 10.3390/jcm10112366 (PMC8199085; doi:10.3390/jcm10112366)

Supplemental Material

Figure S1. Map of (A) Overall Health Rankings, (B) All Outcomes Health Rankings, and (C) All Determinants Health Rankings for 50 States of the US, 1999 to 2018.

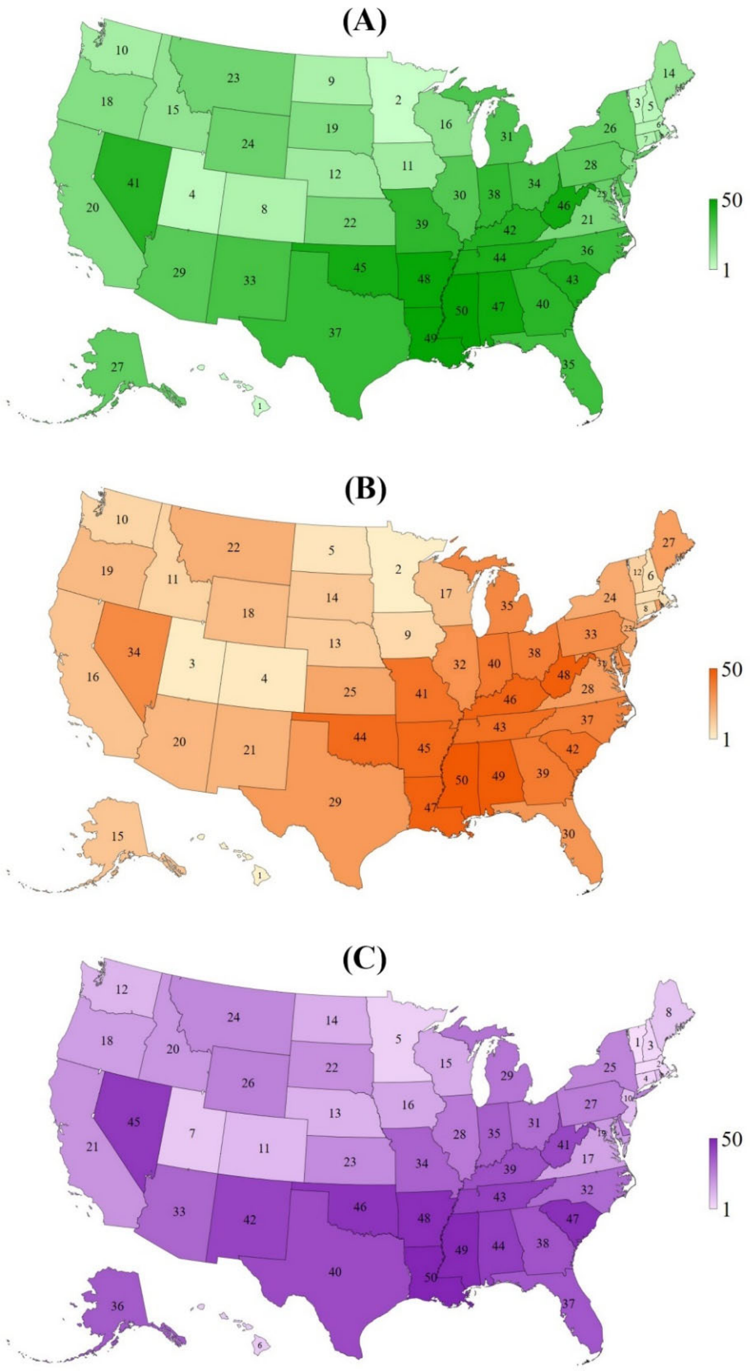

Figure S2. Map of FSR-WASOG Sarcoidosis Clinics for 50 States of the US.

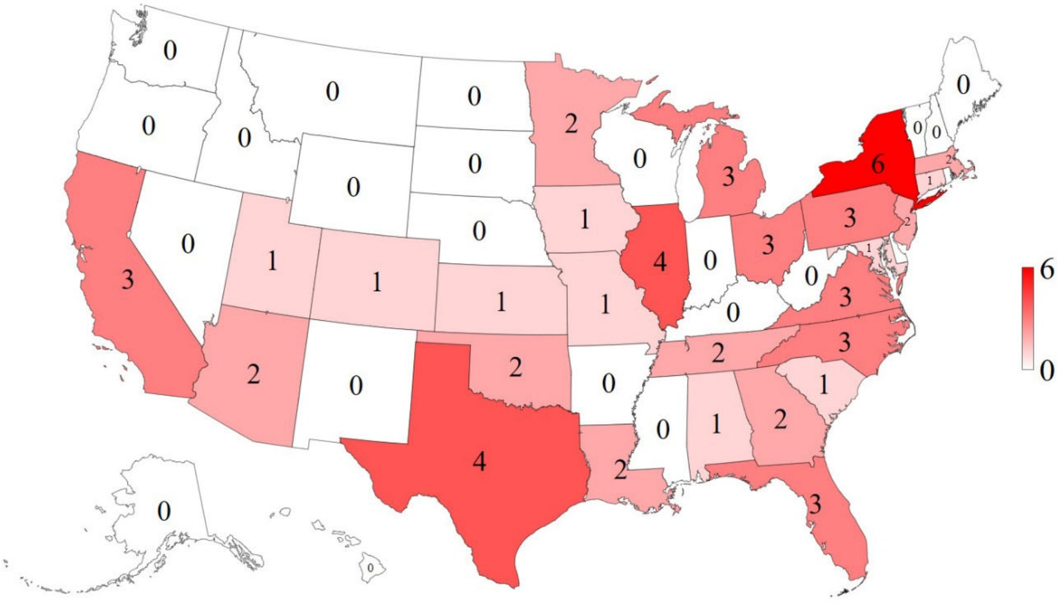

Figure S3. Association Between FSR-WASOG Sarcoidosis Clinics and Sarcoidosis-related AAMR for (A) All Population, (B) African Americans, and (C) European Americans.

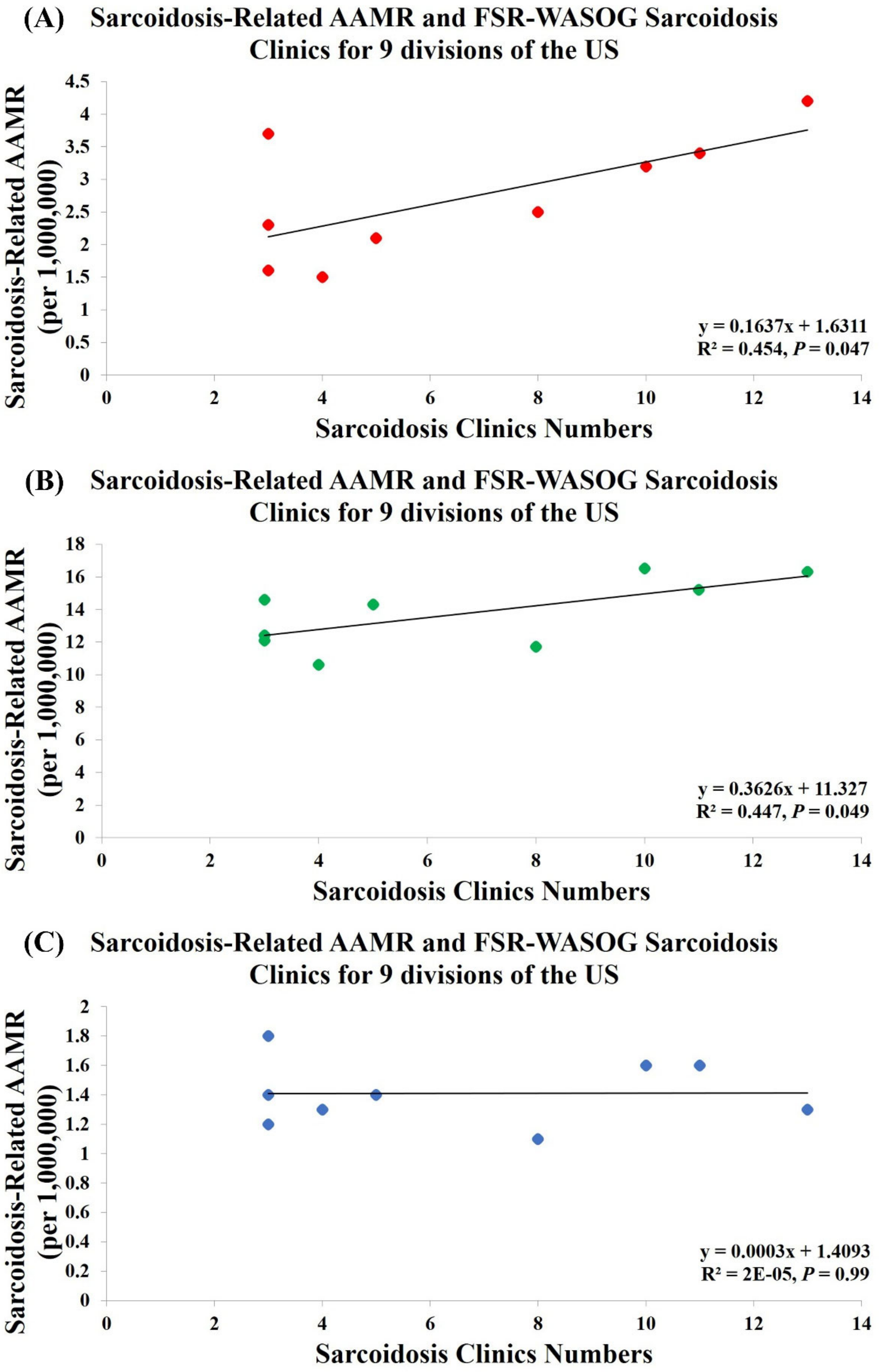

Supplement: Supplementary file 1 [file jcm-10-02366-s001.zip › jcm-1207946-supplementary.pdf]
